# Dentinal Tubule Penetration of an Endodontic Sealer in the Apical Third of Root Canals After Different Final Irrigation Techniques

**DOI:** 10.3390/jcm15030930

**Published:** 2026-01-23

**Authors:** Noelia Santamaria, Jaime Bascones, Carlos Gallego-Garcia, Lucia Gancedo-Caravia

**Affiliations:** 1 Department of Conservative Dentistry and Prosthetics, Faculty of Dentistry, Complutense University of Madrid, 28040 Madrid, Spain; noelsant@ucm.es (N.S.); lgancedo@ucm.es (L.G.-C.); 2 Department of Clinical Dentistry, Faculty of Biomedical Sciences, Universidad Europea de Madrid, 28670 Madrid, Spain; 3 Advanced Light Microscopy Facility, Centro de Biología Molecular Severo Ochoa (CSIC-UAM), 28049 Madrid, Spain; cgallego@cbm.csic.es

**Keywords:** confocal laser scanning microscopy, EndoActivator, conventional irrigation, XP-endo Finisher, ultrasonic activation, tubule penetration, BC Universal sealer

## Abstract

**Background/Objectives**: This study evaluates the penetration of a calcium silicate-based sealer (BC Universal) into dentinal tubules after different final irrigation protocols. **Methods**: Eighty-four single-rooted extracted teeth were instrumented with ProTaper Gold to size F4 and assigned to four groups (*n* = 21) according to the final irrigation protocol as follows: conventional needle irrigation (CNI), sonic agitation with EndoActivator (EA), ultrasonic activation (UA), and XP-Endo Finisher (XPF). A total of 20 canals from each group were filled with BC Universal sealer mixed with fluorescein and the single-cone obturation technique. The remaining specimen in each group served as a negative control to assess potential imaging bias. Specimens were sectioned 3 mm from the apex and analyzed under confocal laser scanning microscopy. Sealer penetration was assessed by penetration area (PA), maximum depth (MaxD), mean depth (MeanD), and percentage of canal perimeter infiltrated (P). Data were analyzed using Kruskal–Wallis or ANOVA tests (α = 0.05). **Results**: All activation/agitation techniques showed significantly higher penetration than CNI across all variables (*p* < 0.001). No significant differences were found among EA, PUI, and XPF for PA, MaxD, and MeanD. However, XPF exhibited the highest perimeter infiltration, which was significantly greater than EA and UA (*p* < 0.001). **Conclusions**: Irrigant activation significantly enhanced dentinal tubule penetration of BC Universal sealer compared to CNI. XPF provided superior P, suggesting superior circumferential distribution. These findings suggest a more effective cleaning of the root canal in the apical third achieved by the tested irrigant activation/agitation techniques, which may improve the sealing potential of BC Universal sealer.

## 1. Introduction

Apical periodontitis is a consequence of bacterial infection of the pulp tissue and the root canal system. The main goal of endodontic treatment is to eliminate these pathogens and prevent further infection [1]. However, prognosis depends on the effectiveness of infection control, which may be difficult to achieve because of the complex root canal anatomy [2]. Its intricate internal morphology hinders the action of instruments, leaving inaccessible areas such as isthmuses, lateral canals, and dentinal tubules untouched [3,4]. These spaces may retain tissue remnants, bacteria, and dentine debris, particularly in the apical portion, where irregularities are more frequently found. As a result, root canal cleanliness may be compromised, the adaptation of filling materials may be limited, and residual substrates may facilitate bacterial recontamination [5].

Irrigation is essential to clean non-instrumented areas and eliminate microorganisms beyond the reach of instrumentation [4]. Conventional needle irrigation (CNI) provides insufficient irrigant penetration into the apical third, especially in untreated areas and complex anatomies [6]. This limitation has led to the development of activation techniques designed to improve irrigant distribution [7]. EndoActivator (EA, Dentsply Sirona, Ballaigues, Switzerland) is a sonic agitation system operating at low frequencies (160–190 Hz) with flexible, non-cutting polymer tips that safely agitate the irrigant while minimizing smear layer production [8]. In contrast, ultrasonic activation (UA), a widely used method [9], operates at higher frequencies (25–30 kHz), generating acoustic streaming and cavitation that enhance debris and bacteria removal from anatomical irregularities. Nevertheless, tip contact with canal walls during UA may cause uncontrolled dentine cutting [10]. More recently, the XP-Endo Finisher (XPF, FKG Dentaire) has been introduced to improve cleaning through mechanical agitation; its size-25, non-tapered NiTi design expands at body temperature, allowing contact with areas untouched by shaping instruments [11].

After chemo-mechanical preparation, an adequate obturation with gutta-percha and sealer is required to achieve a three-dimensional seal of the root canal system. This depends on achieving intimate sealer adaptation to the canal wall dentin [12]. Calcium silicate–based sealers (CSBS) have gained prominence due to their biocompatibility, bioactive behavior, and favorable sealing performance [13]. Their hydration releases calcium ions that interact with dentine, promoting mineral exchange at the sealer–dentin interface, and potentially improving interfacial adaptation [14]. These characteristics make CSBS particularly suited for the single-cone technique, where sealer flow is fundamental for filling canal irregularities [13]. BC Universal Sealer (FKG Dentaire) is a recently introduced CSBS with physicochemical characteristics that meet ISO specifications, showing adequate flow and acceptable film thickness [15].

Although irrigant agitation and activation techniques have demonstrated improved cleaning of the dentinal tubules compared to conventional needle techniques [16], current in vitro evidence remains difficult to compare because of substantial methodological variability [17]. Since sealer penetration contributes directly to obturation quality [5], clarifying how these activation/agitation methods affect the penetration of CSBS is clinically relevant, especially in the apical third, which is the most anatomically complex region and where effective cleaning with irrigants is most challenging [2]. In this context, the apical region deserves special attention, as age-related dentinal sclerosis and reduced tubule permeability, which preferentially develop in the apical portion of the root, may further limit sealer penetration and contribute to the variability reported in previous studies [18]. Accordingly, sealer penetration has been commonly assessed at a standardized apical level, such as 3 mm from the apex, in confocal laser scanning microscopy–based studies evaluating both the depth and circumferential distribution of root canal sealers [19,20,21].

Therefore, this study aimed to compare the effect of EA, UA, XPF, and CNI on the penetration of BC Universal Sealer in the apical third. The null hypothesis was that there is no significant difference in the penetration into dentinal tubules of BC Universal sealer between the experimental groups.

## 2. Materials and Methods

### 2.1. Specimen Selection and Preparation

The study was conducted with the approval of the local ethics committee (C.I. 23/462-E). A total of 84 extracted teeth for orthodontic or periodontal reasons from patients aged 55 to 75, with a single canal, complete root formation, and without caries or fractures, were selected and stored in a 0.1% thymol solution until use, no longer than 2 months. Sample size was calculated based on the data from Eid et al. [22] using G*Power 3.1 (v3.1.9.7, Heinrich-Heine-Universität Düsseldorf, Düsseldorf, Germany) for an ANOVA test with four groups, an effect size of 0.44, a power of 0.9, and an α error of 0.05, yielding 20 specimens per group. An additional specimen was included in each group to serve as a negative control to assess potential imaging bias during confocal microscopy analysis.

Teeth were standardized to a root length of 16 mm by sectioning the coronal end with a cutting machine (Exakt cutting unit 400C, Exakt Advanced Technologies, Norderstedt, Germany). A #10 manual file was introduced in the root canal to prevent blockage, while the external surface of the root was sealed with cyanoacrylate to create a closed system.

The samples were placed in a container filled with water at 37 °C ± 2 °C to simulate body temperature. Root canal preparation was performed with the ProTaper Gold system (Dentsply Sirona, Ballaigues, Switzerland) up to an F4 file using the X-Smart Plus motor (Dentsply Sirona) and irrigated between files with 1 mL of 5.25% sodium hypochlorite (NaOCl, Dentaflux, Madrid, Spain) using a 30-G double-side-vented (DS needle, Dentsply Sirona).

### 2.2. Final Irrigation Protocol: Experimental Groups

The specimens were randomly allocated into four groups (*n* = 21) according to the final irrigation protocol to be used prior to obturation as follows:

Conventional Needle Irrigation (CNI): A 30-G DS needle (Dentsply Sirona) placed 1 mm from the working length (WL) delivered 5 mL of 5.25% NaOCl for 1 min, followed by 5 mL of 17% ethylenediaminetetraacetic acid (EDTA, CanalPro EDTA, Coltene Whaledent, Langenau, Germany) for 1 min.

Sonic Agitation (EA): 5 mL of 5.25% NaOCl was delivered into the canal, and then the EndoActivator device (Dentsply Sirona) with a medium polymer tip (25/04) was activated at 10,000 cycles per minute for 1 min [23]. The procedure was repeated with 5 mL of 17% EDTA.

Ultrasonic Activation (UA): After delivering 5 mL of 5.25% NaOCl into the canal. An ultrasonic tip #25/0.00 (IrriSafe, Satelec/Acteon, Mérignac, France) was placed 2 mm short of the WL and activated at power level 5 for 1 min [24]. The procedure was repeated with 5 mL of 17% EDTA.

XP-endo Finisher (XPF): The canal was irrigated with 5 mL of 5.25% NaOCl, and the XP-endo Finisher file (FKG Dentaire, La Chaux-de-Fonds, Switzerland) was inserted to WL and activated with the X-Smart Plus motor at 800 rpm and 1 N·cm torque for 1 min [25]. The procedure was repeated with 5 mL of 17% EDTA.

Finally, all the canals were irrigated with 5 mL of NaOCl without activation, followed by 5 mL of distilled water, and then dried using ProTaper Gold F4 paper points (Dentsply Sirona). In all groups, the irrigants were applied using a 30-G DS needle inserted 1 mm from the WL.

### 2.3. Root Canal Filling

BC Universal Sealer (FKG Dentaire) was mixed with sodium fluorescein (Alquera, Madrid, Spain) at 0.01% to be used in 20 samples per group. An F4 master cone (Conform Fit, Dentsply Sirona) was used to seal the root canal with the single-cone technique. A total of 0.05 mL of sealer was introduced into the canal using a polymer tip, delivering it from the middle third until it became visible at the canal orifice. The cone was slowly inserted to reach WL, then cut at the canal orifice using the EQ-V system plugger 50.04 (Meta Biomed, Cheongju, Korea) and compacted with a manual plugger (Meta Biomed). A radiograph was taken in mesiodistal and buccolingual views to confirm the correct length and absence of voids. The coronal access was sealed with composite (Ceram.x Spectra ST, Dentsply Sirona).

The remaining samples were filled following the same procedure using plain BC Universal Sealer, without the fluorochrome, which served as a negative control of fluorescent signal.

All procedures were performed by one operator. The samples were then stored at 37 °C and 100% humidity for two weeks to allow the setting of the sealer.

### 2.4. Sample Processing and Analysis

Following the setting period, each root was cut perpendicularly to the longitudinal axis at 3 mm from the apex, using a 0.2 mm cutting blade (Exakt Advanced Technologies, Norderstedt, Germany) with copious cold-water irrigation, to obtain a section 1 mm ± 0.1 mm thick. The coronal surface of the section was polished using fine and superfine-grain Super-Snap disks (Shofu Inc., Kyoto, Japan).

Samples were imaged using a confocal laser scanning microscope (CLSM; Zeiss LSM 900, Carl Zeiss, Jena, Germany) at a wavelength of 488 nm with a resolution of 1024 × 1024 pixels. Images were acquired at both 5× and 20× magnifications to ensure consistent evaluation across different resolutions. Images were captured at 20× magnification with an intensity of 500 V. Z-stacks were captured with 4.5 μm spacing between slices.

**Figure 1:**
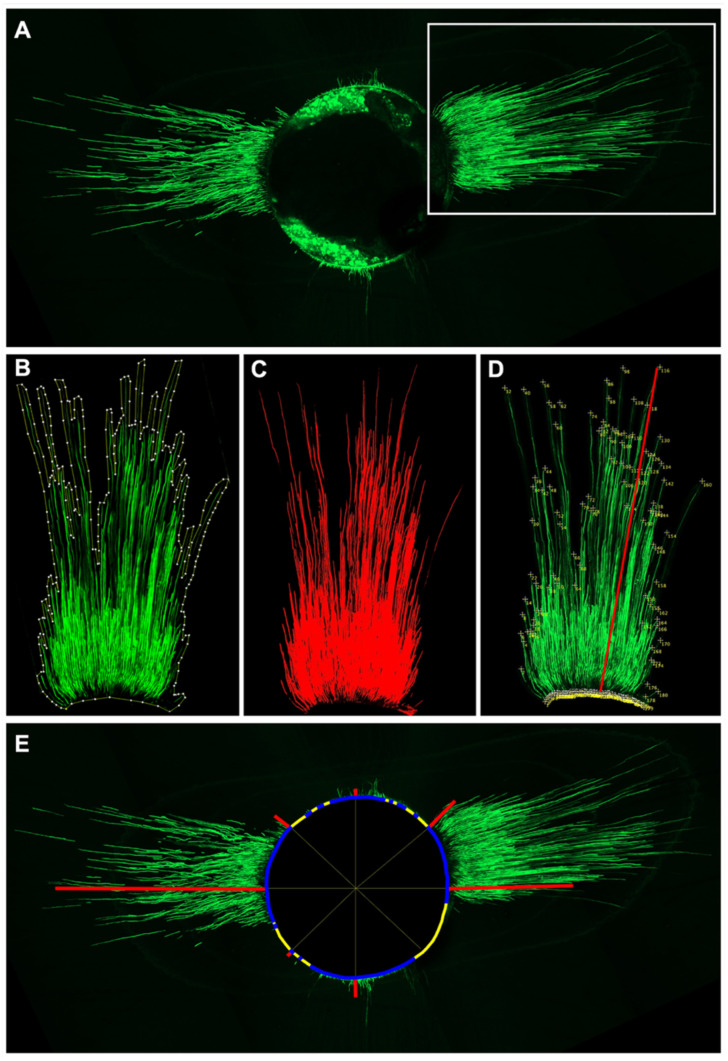
Representative image of the stack after combining all acquired slices showing the green fluorescence of the sealer and the measurements performed to quantify sealer penetration (**A**) maximum intensity projection obtained from the stack. The white rectangle highlights one of the regions of interest to be segmented and analyzed in the following images. (**B**) Enlarged view of the selected region of interest. (**C**) Application of the “MinError” thresholding algorithm to segment the area of sealer infiltration, shown as red overlays for calculation of penetration area (PA). (**D**) Linear measurements along each penetration track were performed using the “Multipoint” tool to calculate MeanD. The red line shows the longest measurement, recorded as MaxD. (**E**) The root canal is divided by 8 radial lines (narrow, yellow) separated by angles of 45° to determine the sealer penetration depth detected on every line for the calculation of 8p-MeanD (represented by red lines); the total length of the root canal perimeter measured using the “Polygon selection” is represented as the combination of thick, yellow, and blue lines. Thick, blue lines show the sections of the root canal perimeter with visible penetration into the dentin wall, identified and measured using the “Segmented Line”, to determine P.

Sealer penetration was quantified using Fiji (ImageJ 2.16.0; National Institutes of Health, Bethesda, MD, USA) through a custom macro designed to automate the measurement process. Three metrics were extracted as follows:

Sealer penetration area (PA): Images were imported using the Bio-Formats Importer and converted into maximum intensity projections (Figure 1A), and the regions of interest were segmented using the “MinError” thresholding algorithm (Figure 1B), which highlights areas of sealer infiltration as red overlays with different pixel intensities for clearer differentiation (Figure 1C). The total sealer penetration area was measured in μm^2^ based on calibrated pixel size.

Maximum sealer penetration depth (MaxD): For each specimen, linear measurements were manually obtained by marking two points along each penetration track, from the canal wall to the deepest visible extent of sealer infiltration, using the “Multipoint” tool in ImageJ (Figure 1D). These lines represented the longitudinal extent of sealer infiltration. The longest of these measurements was recorded as the maximum penetration depth (MaxD) and expressed in μm.

Mean sealer penetration depth (MeanD): calculated by averaging all linear measurements obtained in the previous step across the visible sealer penetration tracks. The resulting mean value was expressed in μm (Figure 1D).

In addition to the previously described measurements, the mean sealer penetration depth was also measured using the method described by Egemen et al. [26]. The root canal was divided by drawing eight radial lines separated by angles of 45°, and the sealer penetration on every line was determined as the distance between the root canal wall and the deepest point of sealer detected. The average of the eight measurements was recorded as eight-point-mean sealer penetration depth (8p-MeanD) (Figure 1E).

The percentage of sealer penetration along the canal perimeter (P) was calculated as follows: The length of the canal wall around the canal was measured using the “Polygon selection” tool and recorded as the total canal perimeter. Then, the perimeter sections where sealer penetration was identified were selected and measured using the “Segmented Line” tool. The lengths of all those sections were added, and the result was divided by the total canal perimeter and multiplied by 100 (Figure 1E).

Measurements were performed by one observer who was blinded to the groups.

### 2.5. Statistical Analysis

Data were analyzed using SPSS version 27.0 (IBM, New York, NY, USA). PA, as well as manual and automated mean penetration depth measurements (8p-MeanD and MeanD), were compared among groups using the non-parametric Kruskal–Wallis test due to the non-normal distribution of the data. The parametric one-way ANOVA test, followed by Tamhane’s T2 post hoc test, was applied for the comparison of MaxD and P. A significance level of 0.05 was set for all statistical tests.

## 3. Results

The samples filled with plain BC Universal Sealer showed no visible sealer infiltration when examined under confocal microscopy. The mean and standard deviation (SD) of the different penetration variables obtained from the test samples are presented in Table 1.

**Figure 2:**
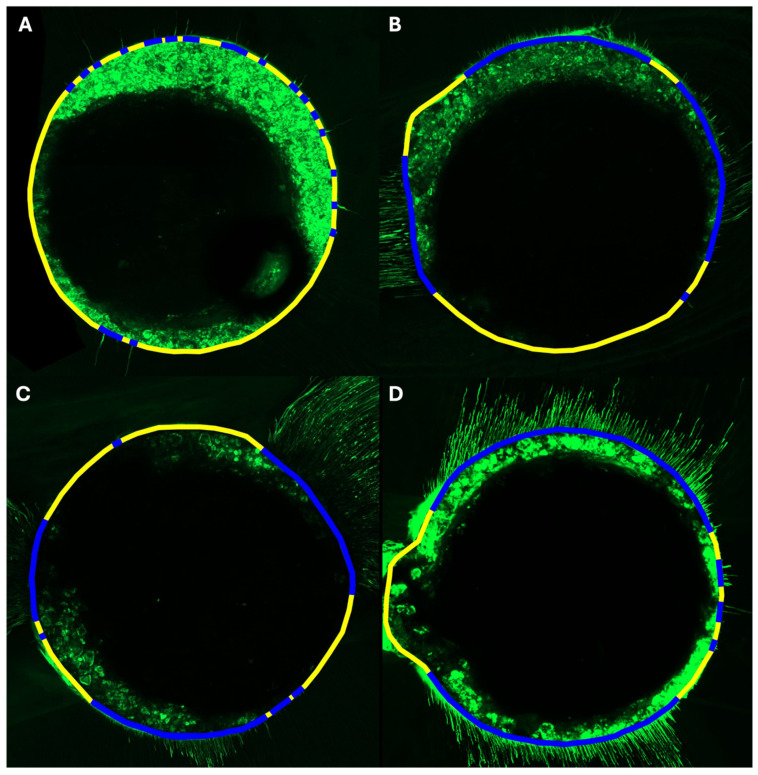
Representative confocal laser scanning microscopy images illustrate the percentage of sealer penetration along the canal perimeter (P) of each group. Green color corresponds to the fluorescent sealer. Blue lines indicate the segments of the root canal perimeter with visible penetration into the dentin tubules, and yellow lines represent the rest of the total perimeter. (**A**) Conventional needle irrigation (CNI), showing 14% perimeter infiltration. (**B**) Ultrasonic activation (UA), showing 51% perimeter infiltration. (**C**) Sonic agitation with EndoActivator (EA), showing 50% perimeter infiltration. (**D**) XP-Endo Finisher (XPF), showing 67% perimeter infiltration.

All agitation/activation techniques (EA, UA, and XPF) demonstrated significantly greater sealer penetration compared to CNI across all measured parameters (*p* < 0.001). PA was significantly higher in EA (mean, 551,759.8; SD, 498,736.2 μm^2^), UA (mean, 470,044.5; SD, 587,526.7 μm^2^), and XPF (mean, 382,055.7; SD, 482.6 μm^2^) groups compared to the CNI group (mean, 8092.9; SD, 5637.6 μm^2^), with no significant differences among the agitation/activation methods (*p* > 0.05). A similar trend was observed for MaxD, where the agitation/activation groups exhibited significantly deeper penetration than CNI (*p* < 0.001), with no significant differences among them. Regarding mean penetration depth, both measuring methods (MeanD and 8p-MeanD) showed significantly higher values in the agitation/activation groups than in the CNI group (*p* < 0.001), again with no differences among techniques. P was significantly greater in all agitation/activation groups compared to CNI (mean, 15.8; SD, 9.8%) (*p* < 0.001). Notably, XPF showed the highest values (mean, 68.8; SD, 17.5%), significantly outperforming both EA (mean, 49.9; SD, 16.7%) and UA (mean, 48.4; SD, 18.2%) (*p* < 0.001). Representative confocal images illustrating the differences in circumferential sealer distribution among the irrigation protocols are shown in Figure 2.

## 4. Discussion

Irrigation protocols have a direct impact on sealer penetration, as they determine the cleanliness and permeability of dentinal surfaces [16], which is crucial in root canal filling to prevent subsequent colonization [5]. This study aimed to compare the penetration of a CSBS sealer in the apical third after different irrigation techniques (EA, UA, and XPF), resulting in significantly greater sealer penetration compared to CNI, leading to the rejection of the null hypothesis.

These results contrast with those reported by Generali et al., who found no statistically significant differences among CNI, EA, and UA [7]. Similarly, Bolles et al. observed that sonic agitation did not significantly enhance sealer penetration [27]. Ates et al. also reported no significant differences among XPF, EA, and CNI [28]. It is important to note that these studies evaluated sealer penetration more apically (1 mm [27] and 2 mm [7,28] from the apex). At these depths, anatomical limitations such as increased dentinal sclerosis and reduced tubule diameter and density [29] may significantly hinder sealer infiltration regardless of the irrigation method used. The present analysis was conducted at 3 mm from the apex, as this sectioning point has been previously used as a reference for the apical third [19,20,21,23]. Nevertheless, it should be acknowledged that this region may offer more favorable anatomical conditions for detecting differences in sealer penetration, since anatomical conditions become progressively more restrictive toward the apical terminus of the root canal. Previous studies have demonstrated a decrease in dentin permeability due to the gradual reduction in dentinal tubule density together with an increase in dentinal sclerosis toward the apical portion of the root [18,29]. Therefore, the penetration patterns observed at 3 mm from the apex in the present in vitro study should not be directly extrapolated to the apical foramen but rather interpreted as representative of sealer behavior under standardized and controlled experimental conditions.

Aksel et al. reported that UA significantly enhanced sealer penetration into dentinal tubules compared to CNI [30]. They observed that UA, along with other protocols, resulted in significantly greater sealer infiltration beyond 100 μm, which highlights the role of irrigant agitation in facilitating deeper tag formation. Similar results have been reported when comparing CNI with UA and XPF [31], although it should be noted that both studies were carried out 5 mm from the apex. Also consistent with this study, higher sealer penetration has been reported with sonic agitation (Endomaster) [16] and UA [32] at 2 mm from the apex and with EA and UA at 3 mm compared to CNI [23].

No significant differences were found between UA and EA in terms of sealer penetration. These findings concur with those reported by Machado et al. [23] and Coşkun et al. [33], suggesting that both techniques may provide comparable ranges of dentinal tubule penetration.

XPF achieved a significantly higher P in the apical third when compared to UA and EA. Contradictory findings have been reported when comparing XPF to other irrigation activation systems. These discrepancies may be related to differences in methodology or the evaluated outcome. Er Karaoğlu et al. focused on irrigant penetration percentages rather than on sealer perimeter coverage and reported superior irrigant penetration percentages with UA compared to XPF at 2 mm from the apex [34]. The present results may be explained by previous findings showing that XPF was more effective in the removal of accumulated hard-tissue debris compared to UA in mesial roots of lower first molars with the presence of isthmuses [11]. These results suggest that the mechanical action of XPF combined with its adaptive design may facilitate greater displacement of debris from the canal walls, allowing a greater circumferential spread of the sealer.

Comparative analyses across the coronal, middle, and apical thirds show less sealer penetration in the apical region [7,27,33]. This has been linked to specific anatomical and histological features of the apical portion: reduced tubule density, occasional absence of tubules, increased sclerosis, and presence of cementum-like material. These characteristics reduce dentin permeability and limit both irrigant and sealer infiltration [29,35]. Furthermore, the apical third is the most inaccessible area to therapeutic procedures, and therefore, it is more susceptible to debris and bacterial persistence [1,5]. Given these challenges, focusing exclusively on the apical third enables a more clinically meaningful evaluation of sealer behavior in the most anatomically complex segment of the root canal.

In addition to anatomical variations, dentin aging may play a significant role in tubule permeability at the apical third. Progressive sclerosis and peritubular dentin formation associated with age cause a significant decrease in tubule density and patency, particularly in the apical third, where these changes are reported to occur more rapidly [18] and often lead to partial or complete tubule obliteration. This fact could explain the internal variability of the results observed in the different groups of the present study, since the selected sample included teeth from donors of a wide range of ages. Taking this aspect into consideration, a comparison of penetration obtained with teeth from different ages should be addressed in the future to establish the impact of aging on sealer penetration.

This study has certain limitations that should be acknowledged. First, only straight, single-rooted canals were included and instrumented to a standardized apical size of 40, which may not represent the anatomical complexity of curved canals or those treated with more conservative instrumentation. Nevertheless, evaluating the apical third of the canal and enlarging its section with wide-diameter instruments allows a more homogeneous sample, as it provides a more round section as well as greater correspondence with the gutta-percha cone [36]. In addition, only one type of sealer was tested, limiting the generalizability of the results to other sealers with different physicochemical properties. Since the aim of the study was to assess the impact of irrigation protocols on the penetration of a CSBS, the single cone obturation technique was selected as the preferred technique to be used with these sealers [37]. Including other sealers, such as AH Plus, which has been associated with the presence of voids when used with the single cone technique [38], may have affected the results, introducing potential interference with the effect of the irrigation protocols. Likewise, warm obturation techniques could have biased the performance of the sealer tested, due to the risk of changes in the material’s physical and chemical properties caused by high temperatures, which should be avoided when using CSBS [37]. With all this in mind, relevant research questions for future studies could be the effect of materials with different characteristics, other obturation techniques, or the inclusion of samples with variable anatomic features, such as curved canals. In addition, analysis with other techniques such as micro-computed tomography [39], scanning electron microscopy, and energy-disperse X-ray spectroscopy [40] could also offer complementary insights into the dentin-sealer interface and help validate correlations across methods.

**Figure 3:**
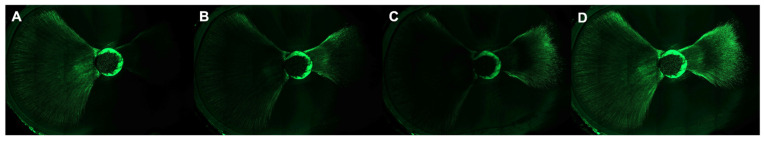
Representative images from the acquired confocal stack. Green color corresponds to the fluorescent sealer. Sequential optical sections captured at different focal depths: (**A**) section at 10 μm from the surface; (**B**) section at 22.5 μm from image (**A**); (**C**) section at 45 μm from image (**A**); (**D**) maximum intensity projection combining all acquired sections in the stack.

A major strength of the present study lies in the deep scanning protocol employed [41]. Rather than relying on a single static image from one focal plane -as is common in previous studies [7,27], multiple optical slices at different depths across each section were acquired, enabling the reconstruction of detailed three-dimensional representations of sealer penetration. This depth-resolved approach offers a more accurate and comprehensive understanding of the variability of penetration throughout the dentin structure. Penetration patterns varied significantly across different depths within the same sample, as shown in Figure 3, underscoring the limitations of single-plane analysis. Overall, this methodology enables a more nuanced interpretation of sealer behavior within the root canal system. As far as the authors are aware, there are only two previous studies that describe in their methodology the superimposition of images obtained at different depths [42,43].

The reliability of CLSM imaging is closely linked to the choice of fluorochrome. Rhodamine B has traditionally been the most widely used; however, recent studies have raised concerns regarding its accuracy, particularly when used with CSBS. It has been reported that rhodamine B may leach from the sealer even after setting and can also diffuse into dentinal tubules or bind to water content, resulting in false-positive signals [44,45]. In the present study, fluorescein was selected as a labeling agent, following its validated use in previous CLSM-based studies [34,39,46,47]. This dye exhibits favorable fluorescence intensity and optical properties and, when properly incorporated, enables reliable visualization without the diffusion-related artifacts associated with rhodamine B. In this case, fluorescein was used at a concentration of 0.01%, which proved sufficient to detect penetration within the dentinal tubules without compromising the integrity of the results or interfering with the physical properties tested.

The present study employed various measurement approaches. Several authors have questioned the clinical relevance and reliability of maximum depth, as it may reflect isolated deep penetration points rather than a uniform distribution [33,48,49]. In contrast, mean depth and penetration area are increasingly recognized as more meaningful indicators of sealer behavior across the canal wall [26,33,50]. The calculation of PA, MaxD, and MeanD was performed using a custom macro developed in ImageJ. This semi-automated tool applies threshold-based segmentation to selectively identify only those regions where sealer is present within the dentinal tubules, effectively excluding non-infiltrated areas—such as intertubular spaces or gaps—typically visualized as dark zones in CLSM images. This allows for an accurate and realistic quantification of the true sealer penetration area, avoiding the overestimations that can result from including unfilled spaces within the measurement. For the calculation of mean depth, previous studies have measured the penetration at certain fixed locations (4 [49], 6 [39], 8 [26], 12 [51], or 32 [42] points). This procedure could overlook greater depths between points. In contrast, this study included two complementary MeanD measurements: one following an eight-point method and another based on the mean depth of all visible sealer-filled tubules throughout each sample, as suggested by Akcay et al. [50].

Performing measurements at different points [26,42,51] has been proposed to minimize the potential bias caused by asymmetric tubule distribution. Irregular patterns could be more noticeable in teeth exhibiting the so-called “butterfly effect”, which is an optical phenomenon often seen in transverse root sections. This anatomical variation is characterized by a higher density of dentinal tubules in the buccolingual direction, potentially asymmetrical irrigant and sealer penetration [51]. For this reason, quantification of penetration can be better established considering the entire perimeter of the root canal, rather than determining maximum and mean depth measurements. With this in mind, another variable was included: P allows a better assessment of the circumferential sealer spread [52]. Although this parameter may overestimate sealer performance in cases where penetration is shallow but continuous [49], it has also been suggested to be a good indicator of the sealer-dentin interface [39].

Taken together, the methodology used allowed for a robust, multi-dimensional evaluation of sealer penetration in the apical third, enhancing both reliability and comparability across activation protocols.

## 5. Conclusions

Within the limitations of this in vitro study, it can be concluded that irrigation agitation/activation techniques improve sealer penetration of BC Universal Sealer into dentinal tubules of the canal wall in straight, single-rooted teeth. Under the standardized experimental conditions applied, XP-Endo Finisher achieved superior circumferential distribution of the sealer at the apical third compared with the other protocols tested.

## Figures and Tables

**Table 1:** Mean and standard deviation (SD) of penetration parameters obtained with the four irrigation protocols.

	Group								*p*	Effect Size (η^2^)
	CNI		EA		UA		XPF			
	Mean	(SD)	Mean	(SD)	Mean	(SD)	Mean	(SD)		
**PA (μm^2^)**	8092.9 ^a^	(5637.6)	551,759.8 ^b^	(49,8736.2)	470,044.5 ^b^	(587,526.7)	382,055.7 ^b^	(482,570.1)	<0.001	0.567
**MaxD (μm)**	76.9 ^a^	(35.8)	994.8 ^b^	(424.8)	912.2 ^b^	(566.6)	743.7 ^b^	(486.2)	<0.001	0.426
**MeanD (μm)**	31.8 ^a^	(20.3)	524.5 ^b^	(299.4)	510.6 ^b^	(431.0)	426.2 ^b^	(351.3)	<0.001	0.522
**8p-MeanD (μm)**	4.7 ^a^	(4.4)	183.4 ^b^	(156.2)	184.0 ^b^	(180.5)	151.7 ^b^	(149.7)	<0.001	0.492
**P (%)**	15.9 ^a^	(9.8)	50,0 ^b^	(16.8)	48.4 ^b^	(18.2)	68.9 ^c^	(17.5)	<0.001	0.600

Different superscript letters indicate statistically significant differences among groups (*p* < 0.05). PA, penetration area; MaxD, maximum penetration depth; MeanD, mean penetration depth; 8p-MeanD, eight-point-Mean sealer penetration depth; P, percentage of perimeter with penetration; CNI, conventional needle irrigation; EA, sonic agitation with EndoActivator; UA, ultrasonic activation; XPF, XP-Endo Finisher file.

## Data Availability

The data that support the findings of this study are available from the corresponding author upon reasonable request.

## Abbreviations

The following abbreviations are used in this manuscript:

CNI	Conventional needle irrigation
EA	EndoActivator
UA	Ultrasonic activation
XPF	XP-Endo Finisher
SD	Standard deviation
CSBS	Calcium silicate–based sealers
WL	Working Length
NaOCl	Sodium hypochlorite
EDTA	Ethylenediaminetetraacetic acid
CLSM	Confocal laser scanning microscope
MeanD	Mean sealer penetration depth
PA	Sealer penetration area
MaxD	Maximum sealer penetration depth
8p-MeanD	Eight-point-mean sealer penetration depth
P	Percentage of sealer penetration along the canal perimeter
